# Preparation of Highly Active Mg-Al-Li-B Alloys via High-Temperature Sintering

**DOI:** 10.3390/ma19020217

**Published:** 2026-01-06

**Authors:** Yuze Wang, Hanqing Xu, Zhihua Zhuang, Jinyan He, Wenlian Peng, Xinggao Zhang, Hao Chen, Liang Zhou

**Affiliations:** Research Institute of Chemical Defense, Academy of Military Sciences, Beijing 102205, China; asciiwdnmd@163.com (Y.W.); hanqing_xu2025@163.com (H.X.); zhihua0802@163.com (Z.Z.); hejinyanfhy@163.com (J.H.); pennwilliam@163.com (W.P.); xinggaozhang@aliyun.com (X.Z.)

**Keywords:** Mg-Al-Li-B alloy, sinter temperature, holding time, combustion heat value, activation energy

## Abstract

Boron is a promising fuel, but its oxide layer impedes combustion. Alloying boron with other high-energy metals can significantly enhance its combustion performance. In this study, we sintered highly reactive lithium-containing Mg-Al-Li-B alloys using magnesium, aluminum–lithium alloy, and boron powder as raw materials. The effects of sintering temperature and holding time on the microstructure were investigated, and the combustion heat value and oxidation resistance of the alloy were tested. Results indicate that sintering temperature significantly influences phase formation: increasing temperature boosts phase content while reducing metallic phases, with 1100 °C identified as the optimal sintering temperature. Holding time had no discernible impact on the phase composition or combustion heat value of the sintered alloy. Alloying enhances material density, thereby increasing volumetric heat value. Thermal oxidation performance tests demonstrate that Li addition significantly lowers the alloy’s oxidation reaction temperature and activation energy, enhancing its reactivity. This high-heat-value, highly reactive alloy holds significant potential for application in pyrotechnics and propellants.

## 1. Introduction

Energetic metal fuels possess characteristics such as high energy density and high mass-specific heat, making them a long-standing key material for solid fuels and combustion agents. Numerous other systems also focus on enhancing specific properties of metallic combustion agents. Typical metal fuels include aluminum (Q = 30.98 kJ/g), magnesium (Q = 24.90 kJ/g), and boron (Q = 58.62 kJ/g). Among these, boron stands out as a material with a high theoretical heat value and low environmental impact. It exhibits rapid combustion rates and high combustion gas pressures at elevated temperatures [1], holding significant potential in fields such as pyrotechnics and propellants. However, boron forms a dense oxide layer during combustion that impedes the reaction, significantly reducing its combustion efficiency.

To address boron’s combustion efficiency issue, it can be combined with other metals or oxidizers. The synergistic effects of combustion between different components can enhance boron’s combustion. Aluminum, characterized by a high energy density and combustion heat value, finds extensive application in energetic materials such as propellants, pyrotechnics, and explosives [2,3,4]. Xu et al. [5] measured the combustion heat of Al-B mixtures using oxygen bomb calorimetry. Results showed pure B exhibits a combustion heat value of 15.6 kJ/g with a combustion efficiency of 26.5%. For Al/B mixed powders (each group representing a 10% increase in B content), the B combustion efficiency reached 97% at Al/B = 90/10, while the highest combustion heat of 38.2 kJ/g was achieved at Al/B = 60/40. Liang et al. [6] tested the ignition combustion performance of mechanically mixed Al-2B powder and Al-2B alloy powder. The results showed that the ignition delay time (56 ms) of the mixed powder was shorter than that of the alloy (102 ms), but the maximum combustion intensity was lower than that of the alloy powder. The combustion duration (79 ms) was shorter than that of the compound powder (198 ms). Additionally, the initial phase of combustion for the Al-2B mixture was unstable, producing numerous sparks and fluctuating combustion intensity. Guo et al. [7] experimentally investigated the oxidation process of MgB_2_, observing that both the exothermic and weight-gain phases occurred between 1200 and 1665 K, which is lower than the 1919 K observed for amorphous B powder. At elevated temperatures, Mg releases vapor that can disrupt the oxide layer forming on particle surfaces, thereby enhancing combustion efficiency [8]. Micro-explosions occur in low-melting-point Mg-containing alloys like Al-Mg and B-Mg [9,10,11]. However, Mg’s theoretical heat value is low, and its substantial addition reduces the material’s overall combustion heat value. Li possesses a theoretical heat value 1.4 times that of Al and 1.7 times that of Mg. Its low melting (453 K) and boiling (1609 K) points, coupled with oxidation rates significantly exceeding those of Al, make it highly prone to micro-explosions during alloy combustion [12]. Liu et al. [13] tested the combustion performance of Al-Li alloys with Li contents ranging from 0 to 3.5 wt.%. Results showed that higher Li content significantly shortened the ignition delay time, with the 3.5 wt.% alloy reducing it by 65% compared to Al particles of the same size. As a promising synergistic combustion element, Li’s low melting and boiling points, coupled with its high reactivity, make it highly likely to assist in breaking down the B oxide layer through combustion heat and vaporization micro-explosions, without significantly reducing the material’s theoretical heat value.

To develop an energetic metal material with a simple preparation process, high stability, and superior performance, in this study, we selected Mg with a low ignition temperature, high combustion efficiency [14], and high heat value; a highly reactive Al-Li alloy; and high-theoretical-heat-value B as raw materials. A Mg(Al-8Li)B_4_ quaternary alloy was prepared via hot reaction sintering. This study primarily analyzed the effects of sinter temperature and holding time on the alloy’s composition and morphology, established a simple and feasible preparation process, and tested the combustion heat value and thermal oxidation characteristics of the alloy.

## 2. Materials and Methods

### 2.1. Materials and Equipment

Amorphous B powder was produced by Beijing Zhongjin Yanxin New Materials Technology Co., Ltd. (Beijing, China), with a particle size of 1–3 μm; Al and Al-8wt.%Li(Al-8Li) alloy powder was produced by Jiangsu Zhiren Jingxing New Materials Research Institute (Nantong, China), with a particle size of 15–20 μm. Spherical atomized Mg powder was manufactured by Tangshan Weihao Company (Tangshan, China), with a particle size of approximately 20 μm.

The phase composition of the sample was analyzed by X-ray diffraction (XRD) method (Cu Kα; 2θ range: 10–90°; scan rate: 10 deg/min) using a Bruker D8 Advance instrument (Bruker, Billerica, MA, USA).

Scanning electron microscopy was used to characterize the particle morphology with a HITACHI SU8020 instrument (Hitachi, Minato, Japan) in secondary electron imaging mode and surface gold spraying of samples.

The powder density test utilized a BetterPyc 380 True Density Analyzer manufactured by Bettersize Instruments (Dandong, China), with argon gas as the test atmosphere. Measurements were performed five times per group.

Oxygen bomb calorimetry was used to measure the combustion efficiency of the powders. A TRHW-7000C oxygen bomb calorimeter produced by Tianrun Technology (Shijiazhuang, China) was used. The powder was sieved through a 100-mesh screen, assembled into a sealed oxygen bomb, and filled with oxygen to 3 MPa. The prepared oxygen bomb was then placed into the oxygen bomb calorimeter to begin the measurement. Measurements were performed five times per group.

Thermogravimetric differential scanning calorimetry (TG-DSC) testing was conducted using NETZSCH STA 499F3/F5 (Selb, Germany) and TA DSC25 instruments (New Castle, DE, USA) to analyze the TG-DSC curves of the material at temperatures ranging from room temperature (RT) to 1400 °C; the heating rate was 10 °C/min, and the testing atmosphere was air.

### 2.2. Preparation Process

Mg, Al, and B powders were weighed in a stoichiometric ratio of 1:1:4 (mass ratio of 24:27:43.2), with weighing errors not exceeding 1%. The mixed raw powder materials were placed in a crucible and compacted. The sample was placed in a sealed tube furnace and protected with argon gas. The sintering process involved heating at a rate of 10 °C/min to 600 °C; holding for 30 min; heating to the specified sintering temperature of 900, 1000, or 1100 °C; holding for 1, 2, or 4 h; and, finally, cooling to room temperature in the furnace.

## 3. Results

After sintering, a thin, white oxide layer formed on the powder surface. This occurred because the purging process did not completely remove air from the crucible. For subsequent experiments, samples with the surface white powder layer scraped off were used. Meanwhile, the interior of the crucible turned black, likely due to partial Mg volatilization at high temperatures.

### 3.1. Effects of Sintering Temperature on Solid-Phase Synthesis Reaction

**Figure 1:**
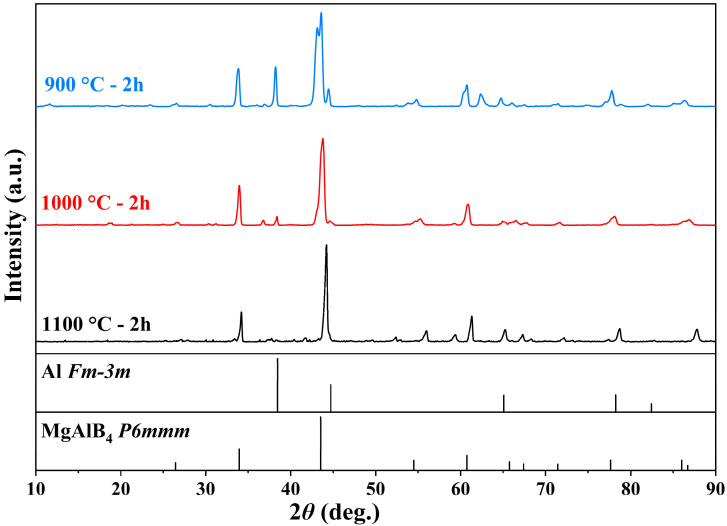
XRD patterns of Mg(Al-8Li)B_4_ powder held at different sintering temperatures for 2 h.

Figure 1 shows the XRD patterns of products sintered for 2 h at different temperatures. Characteristic peaks of MgAlB_4_ appear in the XRD patterns of samples sintered at 900 °C, 1000 °C, and 1100 °C. The intensity of the characteristic peaks of the Al phase decreases with increasing reaction temperature. The sample sintered at 900 °C may have been contaminated, exhibiting a MgO peak. At 1100 °C, the Al peak nearly disappears entirely, indicating that the MgAlB_4_ phase formed at this temperature was the purest.

**Figure 2:**
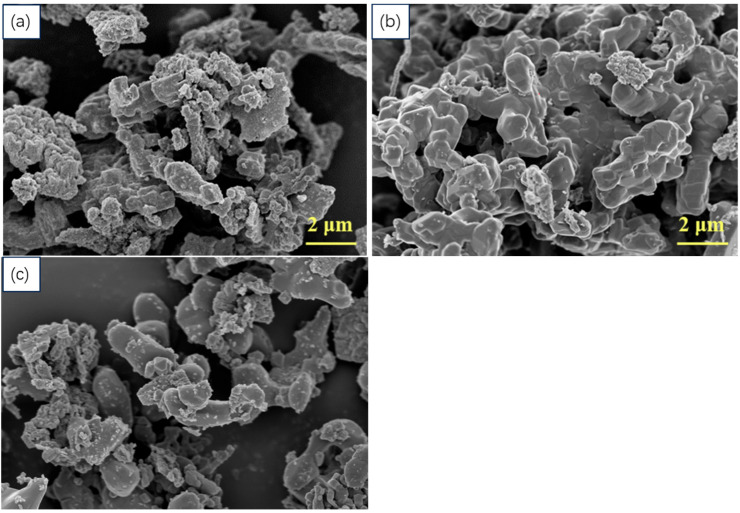
SEM images of Mg(Al-8Li)B_4_ powder after 2 h of holding at different sintering temperatures of (**a**) 900 °C, (**b**) 1000 °C, and (**c**) 1100 °C.

Figure 2 shows the morphology of the alloy powder after holding at different sintering temperatures for 2 h. During sintering, Mg and Al melt into a liquid phase and react with solid boron particles to form new phases. The grains formed by this liquid–solid heterogeneous reaction coalesce together, resulting in irregular powder particle shapes. The surface of the sample sintered at 900 °C exhibits numerous irregular fine particles composed of incompletely reacted, remelted, and recrystallized Al and amorphous B particles [15]. As the sintering temperature increases, the number of fine particles on the powder particle surface decreases, and the powder surface becomes smoother. This is attributed to accelerated interatomic diffusion rates at higher temperatures, which enhance reaction rates and promote more complete reactions. The surfaces of samples sintered at 1000 °C and 1100 °C still exhibit some fine particles, but their morphology is smoother compared to the 900 °C surface particles. This evidence, combined with XRD pattern analysis, suggests that these smooth, fine particles are likely small grains that formed alloy phases but were not fully sintered with the larger particles. To ensure a complete alloy reaction, the Al phase disappears entirely at 1100 °C, confirming this temperature as the subsequent sintering temperature.

### 3.2. Effects of Holding Time on Solid-Phase Synthesis Reaction

**Figure 3:**
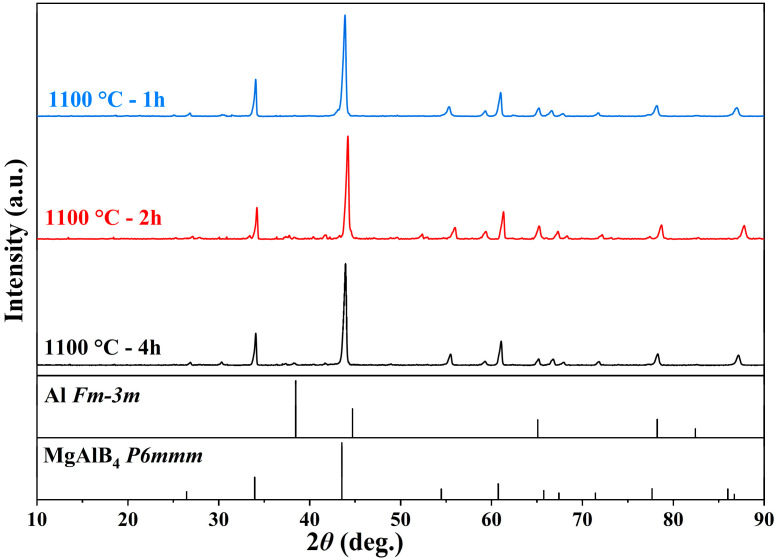
XRD patterns of Mg(Al-8Li)B_4_ powder sintered at 1100 °C with different holding times.

Figure 3 shows the XRD patterns of samples sintered at 1100 °C with different holding times. Considering that excessively long holding times may affect the Mg content in the material, experiments were conducted with holding times of 1 h, 2 h, and 4 h. At 1100 °C, as the holding time increased, the samples all exhibited a pure MgAlB_4_ phase composition, with no significant differences in phase composition. The holding time had no apparent effect on the material’s phase composition.

**Figure 4:**
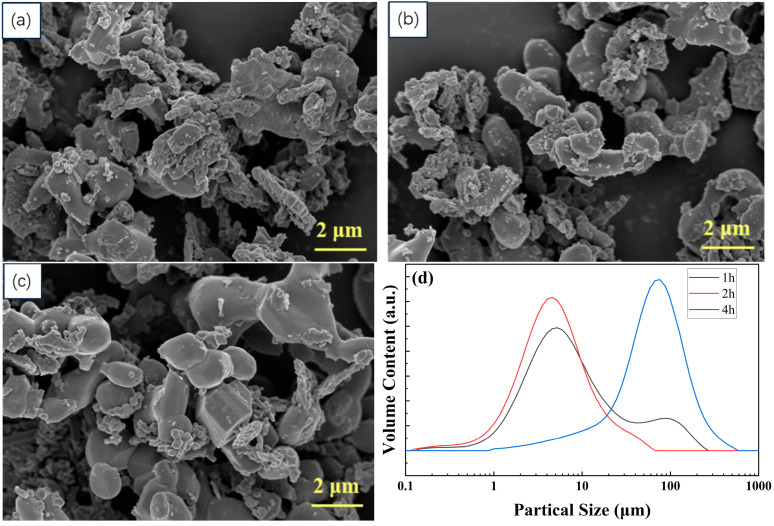
(**a**–**c**) SEM images of Mg(Al-8Li)B_4_ powder sintered at 1100 °C with different holding times of (**a**) 1 h, (**b**) 2 h, and (**c**) 4 h. (**d**) Particle size distribution of Mg(Al-8Li)B_4_ powder.

Figure 4a–c show the morphology of samples sintered at 1100 °C with different holding times. As the holding time increases, the number of fine particles on the sample surface decreases significantly and the particle surfaces become smoother. Figure 4d shows the particle size distribution of the powder at different holding times. After gas-phase dispersion, the particle size distribution was automatically measured using a laser particle size analyzer. The particle size distributions for the 1 h and 2 h holding groups are similar. The bimodal distribution observed in the 1 h group may be attributed to the agglomeration of sintered powder particles. The particle size in the 4 h group increased significantly.

### 3.3. Analysis of Powder Combustion Heat Value and Thermal Oxidation Performance

Table 1 shows the combustion heat values of powders sintered at 1100 °C for different holding times. Prolonging the holding time has no significant effect on the combustion heat value. Alloying increases the powder particle size while forming new phases that raise the energy barrier for oxidation reactions [16]. Although the mass heat value of alloy powders was slightly lower than that of mechanically mixed powders, the density increased after sintering. The alloy density increased by approximately 15.97% relative to the mixed powders, resulting in relative increases in the volumetric heat value of 12.03%, 11.25%, and 11.07% compared to mechanically mixed powders. There was no clear correlation between holding time and the density of the alloy. In applications such as ammunition, missiles, and engine propellants, where space is limited, the volumetric heat value of energetic alloy powders is more critical than their mass heat value. As the holding time increases, the variance in the material’s heat value decreases. This is because longer holding times result in fewer small particles on the surface of the material powder and a more uniform structure, leading to more stable combustion. Considering material morphology and preparation costs, a holding time of 2 h at 1100 °C is determined as optimal.

**Figure 5:**
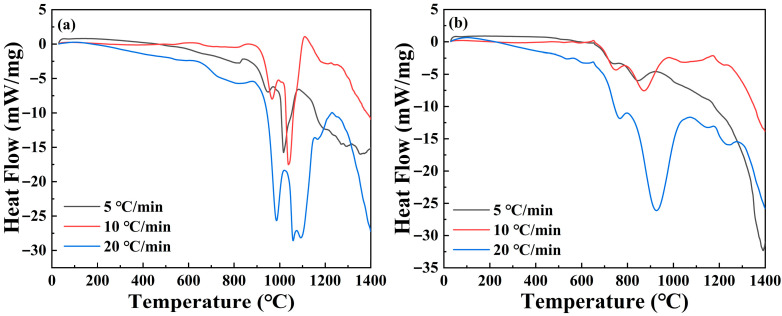
DSC curves of alloy powder at different heating rates from 30 to 1400 °C: (**a**) MgAlB_4_; (**b**) Mg(Al-8Li)B_4_.

Figure 5 shows the TG-DSC curves of MgAlB_4_ and Mg(Al-8Li)B_4_ powders prepared by holding [17,18] at 1100 °C for 2 h, recorded in air at heating rates of 5, 10, and 20 °C/min. The peak temperatures of the maximum exothermic peaks for MgAlB_4_ increased as the heat was increased to 1017.20, 1039.00, and 1059.40 °C. The activation energy of the samples was calculated using the Kissinger method [17,18]. Substituting the data into the equation yielded an activation energy of approximately 334.3 kJ/mol. The peak temperatures of the maximum exothermic peaks for Mg(Al-8Li)B_4_ were 844.62, 874.02, and 927.21 °C, representing reductions of 16.97%, 15.88%, and 12.48% relative to MgAlB_4_. The activation energy was approximately 156.9 kJ/mol, corresponding to a decrease of 53.07% compared to MgAlB_4_. Li doping significantly reduced the oxidation temperature and activation energy, accelerating the oxidation reaction process. Compared to the Li-free MgAlB_4_ alloy, Mg(Al-8Li)B_4_ exhibited higher reactivity and faster oxidation rates, markedly enhancing the alloy’s thermal oxidation performance.

## 4. Discussion

High-temperature sintering of alloy powders primarily involves three stages: preliminary grain rearrangement, the heating stage, and the high-temperature holding stage. During pre-sintering at 600 °C, atoms begin diffusing between solid-phase powder particles, forming sintering necks and reducing interparticle voids. In the heating stage, Mg and Al-Li alloys commence melting, filling voids and uniformly diffusing to encapsulate solid-phase boron particles. During the high-temperature holding stage, solid–liquid heterogeneous reactions occur between Mg, Al-Li alloys, and B, generating alloy phases.

Theoretically, longer holding times lead to more complete reactions and higher densities. However, excessively prolonged holding times increase particle size and accelerate Mg and Li volatilization, reducing the specific surface area and high-activity components, thereby degrading the alloy’s ignition and combustion performance.

Synergistic effects refer to phenomena in combustion whereby highly reactive components undergo oxidation first, releasing heat that is absorbed by less reactive substances, thereby promoting their subsequent combustion. In mechanically mixed powders, Mg, Al, Li, and B lack strong bonding, causing Mg and Al to burn first while the reaction temperatures of the other three components remain relatively independent. Sintering Mg-Al-Li-B into an alloy enhances synergistic effects between components, stabilizing and improving the controllability of combustion heat release. However, this also increases the energy barrier and raises the temperature at which oxidation reactions release heat.

Since the sintering temperature of the alloy exceeds the melting points of all components, except B, molten Mg, Al, and Li rapidly diffuse and distribute uniformly during sintering and holding. This forms the MgAlB_4_ alloy phase, unifying the powder’s oxidation exothermic peak into the characteristic exothermic peak of the alloy phase rather than separating oxidation reactions occurring at distinct temperatures for each reactant with varying reactivity. Simultaneously, the Li distribution within the alloy should be relatively uniform rather than confined solely to the Al-Li alloy phase. This prevents the appearance of exothermic peaks of Li oxidation in the 300–500 °C range for the sintered alloy powder, instead lowering the overall peak temperature. The highly reactive Li element reduces the oxidation reaction temperature without compromising MgAlB_4_ phase formation.

## 5. Conclusions

This experiment investigated a novel preparation process for a metallic fuel, determining the optimal sintering temperature and reaction time.

1. The sintering temperature significantly influences the phase formation of the Mg(Al-8Li)B_4_ alloy. As the sintering temperature increases, the metallic Al phase gradually diminishes until it disappears, while the purity of the MgAlB_4_ alloy phase increases. Unreacted boron particles on the sample surface decrease. To ensure complete reaction of the components in the alloy phase, a sintering temperature of 1100 °C was selected.
2. The holding time has little effect on the phase composition of the alloy. At 1100 °C, the phase composition of the alloy shows no significant difference across various holding times, and the average heat value of the material remains stable. However, as the holding time increases, the number of fine particles on the grain surface decreases, the variance in the material heat value gradually reduces, and the combustion process becomes more stable. An excessive holding time will cause the powder particle size to increase excessively. Considering both preparation cost and material performance, the optimal holding time was determined to be 2 h.
3. The mass heat value of the alloy powder was slightly lower than that of the mixed powder. However, due to increased material density after sintering and alloying, the volumetric heat value of the alloy powder significantly increased. Comparing TG-DSC results of Mg(Al-8Li)B_4_ with added Li and MgAlB_4_ without Li under identical processing conditions reveals that Li addition significantly lowers the peak temperature of the oxidation reaction exotherm, advancing the onset of oxidation. Concurrently, it reduces the material’s activation energy for reaction and enhances its reactivity.

Subsequent preparations of Mg(Al-xLi)B_4_ alloys with varying Li contents will be investigated using the same process. These alloys will undergo tests for combustion heat value, oxidation characteristics, and ignition/combustion performance. The combustion characteristics of Mg(Al-xLi)B_4_ will be analyzed using a tandem system integrating the combustion heat value, reactivity, emission spectroscopy, and flame imaging.

## Figures and Tables

**Table 1:** Combustion heat value of mg(al-8li)b_4_ powder sintered at 1100 °C with different holding times.

Sample	Density (g·cm^−3^)	Mass Heat Value (kJ·g^−1^)	Volumetric Heat Value (kJ·cm^−3^)	Combustion Efficiency (%)
1h	2.77	30.60 ± 1.55	85.32	64.98
2h	2.76	30.70 ± 1.33	84.73	64.76
4h	2.76	30.65 ± 1.20	84.59	64.66
Mix	2.38	32.10 ± 2.17	76.16	67.51

## Data Availability

The original contributions presented in this study are included in the article. Further inquiries can be directed to the corresponding authors.
